# Intraoperative tranexamic acid administration in cranial meningioma surgery: a meta-analysis of prospective randomized, double-blinded, and placebo-controlled trials

**DOI:** 10.3389/fonc.2024.1464671

**Published:** 2024-08-29

**Authors:** Martin Vychopen, Felix Arlt, Erdem Güresir, Johannes Wach

**Affiliations:** ^1^ Department of Neurosurgery, University Hospital Leipzig, Leipzig, Germany; ^2^ Comprehensive Cancer Center Central Germany, University Hospital Leipzig, Leipzig, Germany

**Keywords:** blood loss, complications, meningioma, Tranexamic Acid, transfusion

## Abstract

**Objective:**

Cranial meningioma surgeries often involve significant blood loss and transfusions. Tranexamic acid (TXA) has been used to reduce blood loss in various surgeries. This meta-analysis of randomized placebo-controlled trials (RCTs) evaluates the impact of TXA in cranial meningioma surgery.

**Methods:**

Pubmed, Web of Science, and Cochrane Library were searched for RCTs. Studies were compared for: Blood loss, operative time, hospital stay, reoperation rates, allogeneic and autologous transfusion, and incidence of complications.

**Results:**

Seven RCTs with 490 patients receiving TXA and 491 receiving placebos were included. TXA significantly shortened operative time (Mean Difference (MD): -20.95; 95%CI: -39.94 to -1.95; p=0.03). Blood loss was lower with TXA (MD: -262.7 ml; 95%CI: -397.6 to -127.8; p=0.0001). Odds of reoperation were not significantly different (OR: 0.44; 95%CI: 0.13-1.45; p=0.18). TXA significantly reduced the need for RBC transfusions (OR: 0.47; 95%CI: 0.22-0.99; p<0.05). No significant differences were observed regarding postoperative seizures (OR: 1.06; 95%CI: 0.56-2.03; p=0.85), hydrocephalus (OR: 0.25; 95%CI: 0.03-2.29; p=0.22), or hematoma (OR: 0.52; 95%CI: 0.22-1.28; p=0.16). Hospital stay was shortened in the TXA group (MD: -1.23; 95%CI: -2.41 to -0.05; p=0.04).

**Conclusion:**

This meta-analysis suggests that a single intraoperative dose of TXA reduces blood loss, allogeneic blood transfusions and shortens surgery time.

## 1 Introduction

Tranexamic acid (TXA) is an antifibrinolytic agent developed by Japanese research couple Shosuke and Utako Okamoto shortly after Second World (1). They aimed to control postpartum hemorrhage, which was one of the main causes of maternal death in post-war Japan. Since then, TXA has been adopted in several surgical and non-surgical disciplines as an effective and safe method to enhance hemostasis.

TXA is a lysin derivate that binds to plasminogen, preventing enzymatic degradation of formed fibrin meshwork and stabilizing the formed clot (2). However, up until 2010, controlled studies on TXA use were scarce. The WOMAN study confirmed the reduction of bleeding-associated complications nearly 50 years after the clinical introduction of TXA (3). Subsequently, the CRASH-2 trial proved a significant reduction in bleeding-associated deaths in patient after traumatic injury (4). Blood loss, and subsequent RBC transfusion are known risk factors for intra- and postoperative complications during the treatment course for intracranial meningiomas (5, 6). Furthermore, increasing age and use of anticoagulants presents an additional risk factor, for which the perioperative or even prophylactic use of tranexamic acid might be the solution (7). The resection of meningiomas is mostly performed in an elective surgical setting, which has to aim for most strict and tailored safety profile for the individual operation. To date, several retrospective and cohort studies report on use of TXA in meningioma surgery, usually with mixed results (8). This might be due to strong limitation in contemporary studies, including wide differences in dosing scheme of the TXA, ranging from weight-adjusted doses to standardized single doses of 1g TXA intravenously (9, 10). One of the best data sources on administration scheme comes from the CRASH-2 trial, which suggests that the time between bleeding and application of TXA acid should not overreach 3 hours (4). Apart from venous thromboembolism, TXA is reported to increase the risk of postoperative seizures, and might be a major drawback for the usage of TXA” (11, 12). The present meta-analysis of 981 patients from seven randomized placebo-controlled, and double-blinded studies aims to evaluate the exact risk-benefit ratio of TXA use in cranial meningioma surgery.

## 2 Methods

### 2.1 Search strategy and screening

**Figure 1:**
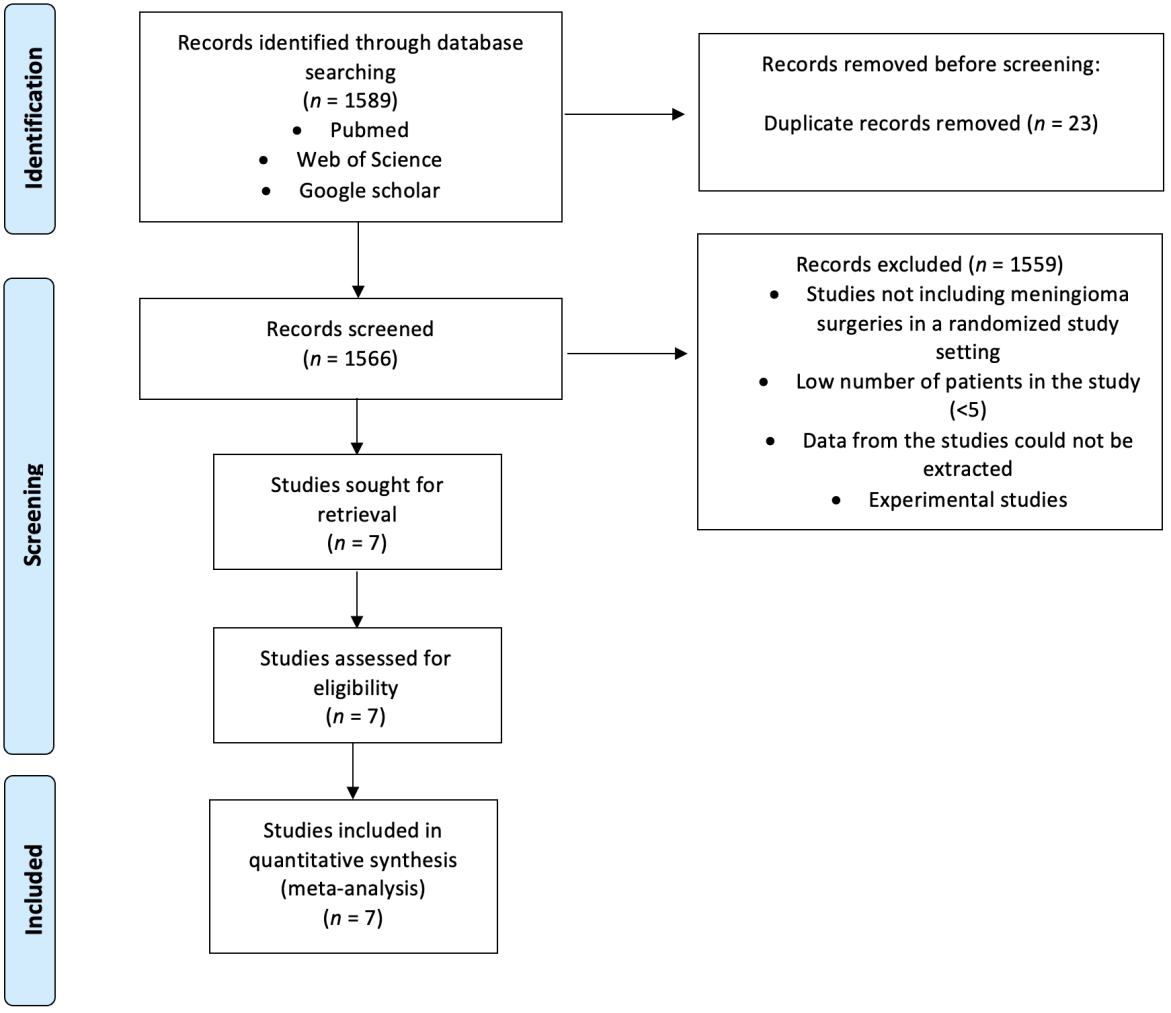
PRISMA flowchart summarizing identification process of included studies.

Following the Preferred Reporting Items for Systematic Reviews and Meta-Analyses (PRISMA) guidelines and the Cochrane Handbook for systematic Review of Interventions Version 6.4, we queried the PubMed, Google Scholar, and Web of Science databases from their inception to May 30, 2024 (13, 14). The search focused on English-language, full-text randomized controlled trials (RCTs) related to tranexamic acid (TXA) usage during meningioma resection using the mesh terms (“tranexamic acid” OR “TXA”) AND (“meningioma”) (see **Figure 1**). After duplicate removal, titles and abstracts were screened using the Rayyan Intelligent Systematic Review web application to identify studies that met the inclusion/exclusion criteria. Two reviewers (J.W. and M.V.) independently screened all studies, with disagreements resolved by a third author (E.G.).

### 2.2 Inclusion criteria

The inclusion criteria were determined according to the PICOS (population, intervention, comparator, outcomes, and study design) framework (15). These criteria were formulated as follows: patients had undergone surgical treatment for cranial meningioma; perioperative tranexamic acid therapies were performed; results were compared to a placebo control; all results of the prespecified endpoints were reported; and the studies were defined as prospective randomized, placebo-controlled, and double-blinded studies.

### 2.3 Data extraction and quality evaluation

Data extracted from the selected studies included publication year, author institution and country, study design, sample size, patient demographics (sex and age), extent of resection, complications (thromboembolic events, seizures, new onset neurological deficits, hematoma, reoperation, hydrocephalus), operative duration, TXA dosage, route and timing of TXA administration, estimated blood loss (EBL), cell saver use, need for blood transfusions [Fresh frozen plasma (FFP) transfusion, Red blood cell transfusion (RBC transfusion), platelet transfusion (PT)], complications (hematoma, hydrocephalus, seizure, revision surgery, venous thromboembolism) and length of hospital stay. Patients’ physical status at discharge was assessed using the Extended Glasgow Outcome Scale (GOSE) and categorized as good recovery (GOSE 7-8), moderate disability (GOSE 5-6), and severe disability (GOSE 1-4) (16). The Cochrane Bias Risk Tool was used to analyze the risk of bias in the included trials using the software Review Manager Web (RevMan Web Version 5.4.1 from The Cochrane Collaboration). The following five characteristics regarding risk of bias evaluation were considered in the analysis: selection bias, performance bias, detection bias, attrition bias, and reporting bias. Finally, a risk of bias summary chart and plot were generated. The National Institutes of Health Quality Assessment Tool for interventional studies (NIH-QAT) was used for the assessment of quality and risk of bias of included studies (17).

### 2.4 Statistical analysis

Statistical analyses were performed on operative duration, complications, transfusion requirements, estimated blood loss (EBL), postoperative seizures, postoperative hematomas, postoperative hydrocephalus, postoperative physical status, and length of hospital stay using Cochrane’s RevMan 5.4 (The Nordic Cochrane Center, Cochrane Collaboration). To assess statistical heterogeneity and inconsistency, χ² and I² statistics were employed, with an I² value of 50% or more indicating substantial heterogeneity (18). The relative contribution of individual studies, based on sample size, was considered in the estimation of treatment effects. To assess publication bias, we created Funnel plots for a visual examination of publication bias in the included studies. Effect sizes of categorical data were expressed as pooled odds ratio (OR) estimates. The relative contribution of individual studies, determined by sample size, was considered in estimating treatment effects. Random effects models were used to create forest plots showing the pooled estimates. This statistical stepwise workflow was applied to investigate the following endpoints: Seizure, need for transfusion (FFP, platelet concentrate, red blood cell concentrates), estimated blood loss, cell saver use, length of hospital stays, operative duration, hematoma, reoperation, hydrocephalus, and GOSE status at discharge.

## 3 Results

### 3.1 Study selection

An initial search of PubMed, Web of Science, and Google Scholar databases identified 1,589 records. After removing 23 duplicate records, 1,566 unique records were screened by title and abstract. Of these, 1,559 records were excluded for reasons such as not including meningioma surgeries in a randomized study setting, having a low number of patients in the study (<5), being unable to extract data from the studies, or being experimental studies. This left 7 studies sought for retrieval and assessed for eligibility. Ultimately, all 7 studies met the inclusion criteria and were included in the quantitative synthesis for the meta-analysis.

The selected studies were randomized, blinded, and placebo-controlled trials, providing primary clinical data on the use of tranexamic acid (TXA) during resection of intracranial meningiomas. The trials were conducted in various institutions and countries, offering a comprehensive overview of TXA’s efficacy and safety in this context. The detailed flowchart of the literature review process is presented in **Figure 1**.

### 3.2 Surgical characteristics of studies included in this meta-analysis

**Table 1** summarizes the characteristics of seven studies included in the meta-analysis on the use of tranexamic acid (TXA) in meningioma surgeries, encompassing a total of 981 patients (19–25). The studies span across various countries including India, Indonesia, Tunisia, Pakistan, and China, with sample sizes ranging from 30 to 600 patients. The mean ages of participants in the TXA and control groups are fairly similar within each study, generally ranging from the late 30s to early 50s. Tumor location data is variably reported, with some studies specifying the proportion of skull base and non-skull base tumors. WHO grades are not consistently reported across the studies. Tumor sizes are provided in five of the seven studies (19, 22–25). The administration of TXA commonly involved a 20 mg/kg preoperative dose over 20 minutes, followed by a maintenance dose of 1 mg/kg per hour intraoperatively, except for Siddiqui et al. (20) who administered a single 2 g preoperative dose. Transfusion triggers vary, with one study specifying criteria such as hematocrit below 27% or hemoglobin below 8 g/dL, while others did not report this information (22, 23). This comprehensive table highlights the diverse methodologies and patient demographics in studies examining TXA’s efficacy and safety in meningioma surgeries.

**Table 1:** Characteristics of the included studies.

Authors & Year	Country	Sample Size (TXA/no TXA)	Mean Age, yrs (TXA/no TXA)	Tumor Location in the TXA Cohort (skull base/non-skull base)	WHO Grade	Tumor Size (TXA vs no TXA)	TXA Dose & Administration Period	Transfusion Trigger
Hooda et al., 2017 (18)	India	60 (30/30)	39.3/41.6	14/16	Not reported	99 mm³ (range 33–387) vs 79 mm³ (range 25–960)	20 mg/kg pre-op over 20 mins followed by 1 mg/kg/hr intra-op	At the discretion of the anesthesiologist
Siddiqui et al., 2018 (19)	India	100 (50/50)	50.1/49.4	Not specified	Not reported	Not reported	2 g pre-op	Not reported
Sutanto et al., 2019 (20)	Indonesia	40 (20/20)	47.4/44.2	Not specified	Not reported	Not reported	20 mg/kg pre-op over 20 mins followed by 1 mg/kg/hr intra-op	Not reported
Ravi et al., 2021 (21)	India	30 (15/15)	48.9/52	6/9	Mostly Grade I	63.79 mm³ (SD 31.11) vs 43.46 mm³ (SD 24.32)	20 mg/kg bolus pre-op followed by 1 mg/kg/hr intra-op	Hematocrit < 27%
Rebai et al., 2021 (22)	Tunisia	91 (45/46)	49.5/48.2	7/38	Not reported	5.05 cm³ (SD 1.07) vs 5.30 cm³ (SD 1.23)	20 mg/kg pre-op over 20 mins followed by 1 mg/kg/hr intra-op	Hemoglobin < 8 g/dL
Khalid et al., 2023 (23)	Pakistan	60 (30/30)	40.4/43.2	2/28	Not reported	Mean 6.13 cm³ (SD 1.51) for both groups	20 mg/kg pre-op over 20 mins followed by 1 mg/kg/hr intra-op	Not reported
Li et al., 2024 (24)	China	600 (300/300)	53/53	159 convexity in TXA/others not specified	TXA: 1&2: 293, 3: 7	3.0 cm (IQR: 2.3–4.2) vs 3.1 cm (IQR: 2.2–4.1)	20 mg/kg pre-op over 20 mins	NICE guideline 2015

### 3.3 Risk of bias and quality assessment

**Figure 2:**
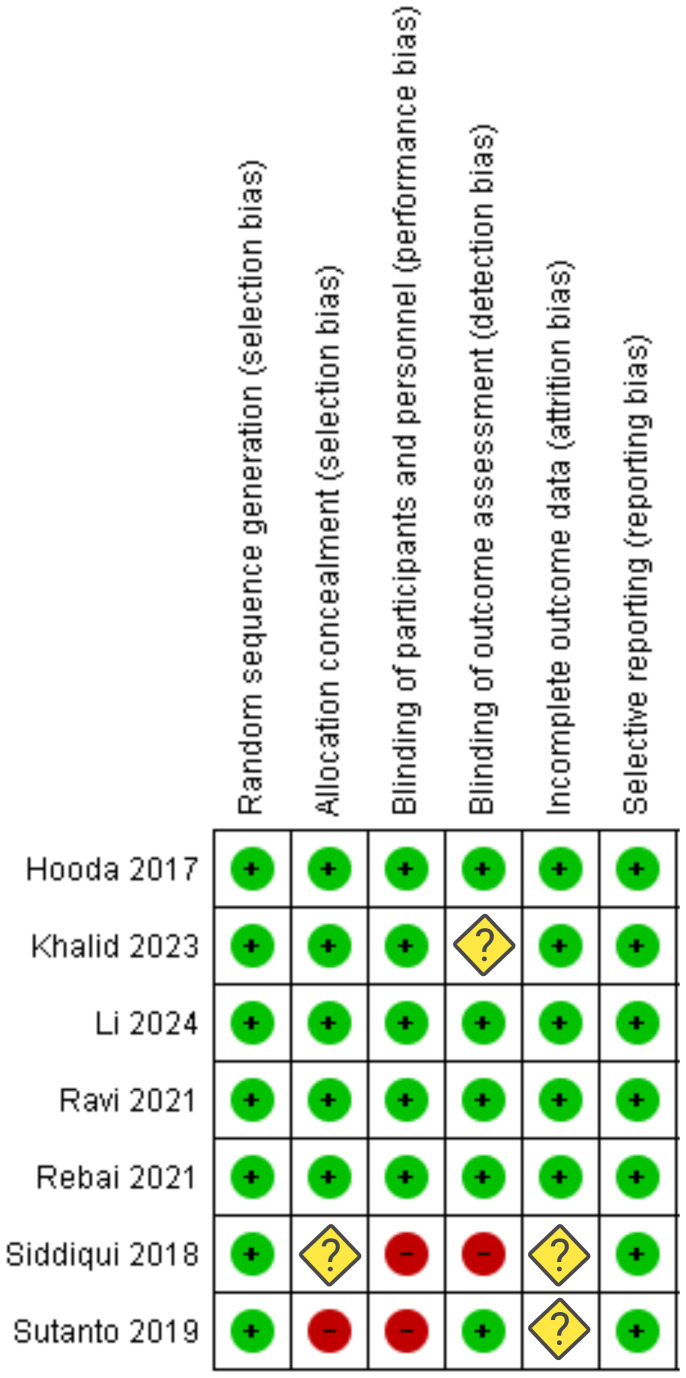
Risk of bias assessment for each category of bias (“+” constitutes low risk; “?” constitutes unclear risk; “-”constitutes high risk).

The risk of bias summary assessed the seven included studies for various biases (see **Figure 2**). All studies were evaluated for random sequence generation, allocation concealment, blinding of participants/personnel, blinding of outcome assessment, incomplete outcome data, and selective reporting. Most studies demonstrated a low risk of bias, indicated by green circles with plus signs. Some studies had unclear risks (yellow diamonds with question marks) or high risks (red circles with minus signs). Specifically, Siddiqui et al. (20) and Sutanto et al. (21) showed high risk or unclear risk in several domains, notably in blinding and random sequence generation. Furthermore, NIH-QAT tool was used to evaluate quality of the included trials (see **Supplementary Figure S1**).

### 3.4 Blood Loss, allogeneic transfusion, and autotransfusion via cell saver

**Figure 3:**
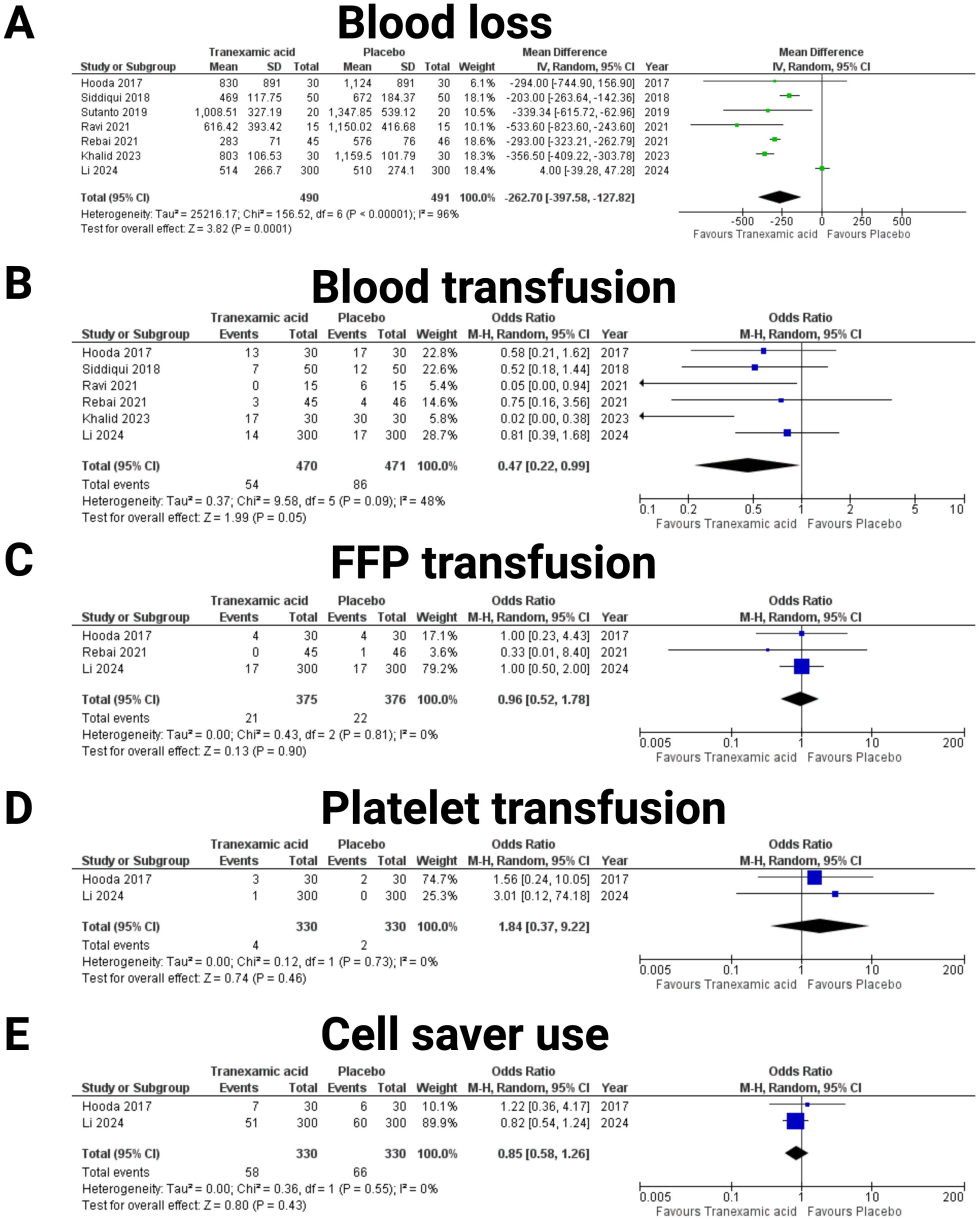
Forest plots illustrating the effect of tranexamic acid versus placebo on various blood loos, allogeneic and autotransfusion. Each panel displays the odds ratio (OR) or mean difference (MD) with 95% confidence intervals (CIs) for individual studies and the combined estimate. The following endpoints were investigated: **(A)** Blood loss, **(B)** Blood transfusion, **(C)** Fresh frozen plasma (FFP) transfusion, **(D)** Platelet transfusion, and **(E)** Cell saver use.

Data from 981 patients out of seven studies were included in the investigation of blood loss (19–25) (see **Figure 3A**). The pooled mean difference in blood loss between the TXA and placebo groups was -262.70 mL (95% CI: -397.58 to -127.82 mL), favoring TXA with a statistically significant reduction in blood loss (*p* = 0.0001). The effect of TXA on the need for blood transfusions was investigated (see **Figure 3B**). Data from six studies were included, comprising a total of 470 patients in the TXA group and 471 in the placebo group (19, 20, 22–25). The combined OR was 0.47 (95% CI: 0.22-0.99), indicating a significant reduction in transfusion requirements with tranexamic acid (*p* = 0.05). The heterogeneity among the studies was moderate (I² = 48%, *p* = 0.09). Furthermore, the impact of TXA on the administration of fresh frozen plasma (FFP) was analyzed (see **Figure 3C**). Data from three studies were included, with 375 patients in TXA group and 376 in the placebo group (19, 23, 25). The combined OR was 0.96 (95% CI: 0.52-1.78), showing no significant effect of tranexamic acid on FFP use (*p* = 0.90). Heterogeneity was low (I² = 0%, *p* = 0.81). The need for platelet transfusion was investigated based on two studies, including 330 patients in the TXA group and 330 the placebo group (19, 25) (see **Figure 3D**). The pooled OR was 1.84 (95% CI: 0.37-9.22), indicating no significant difference in platelet transfusion requirements between the TXA and placebo groups (*p* = 0.46). Heterogeneity was low (I² = 0%, *p* = 0.73). The influence of TXA on the use of cell saver was analyzed from data of two studies (19, 25), with a total of 330 patients in the TXA group and 330 in the placebo group (see **Figure 3E**). The combined OR was 0.85 (95% CI: 0.58-1.26), suggesting nonsignificant reduction in cell saver use with tranexamic acid (*p* = 0.43). Heterogeneity was low (I² = 0%, *p* = 0.55).

### 3.5 Operative time and length of hospital stay

**Figure 4:**
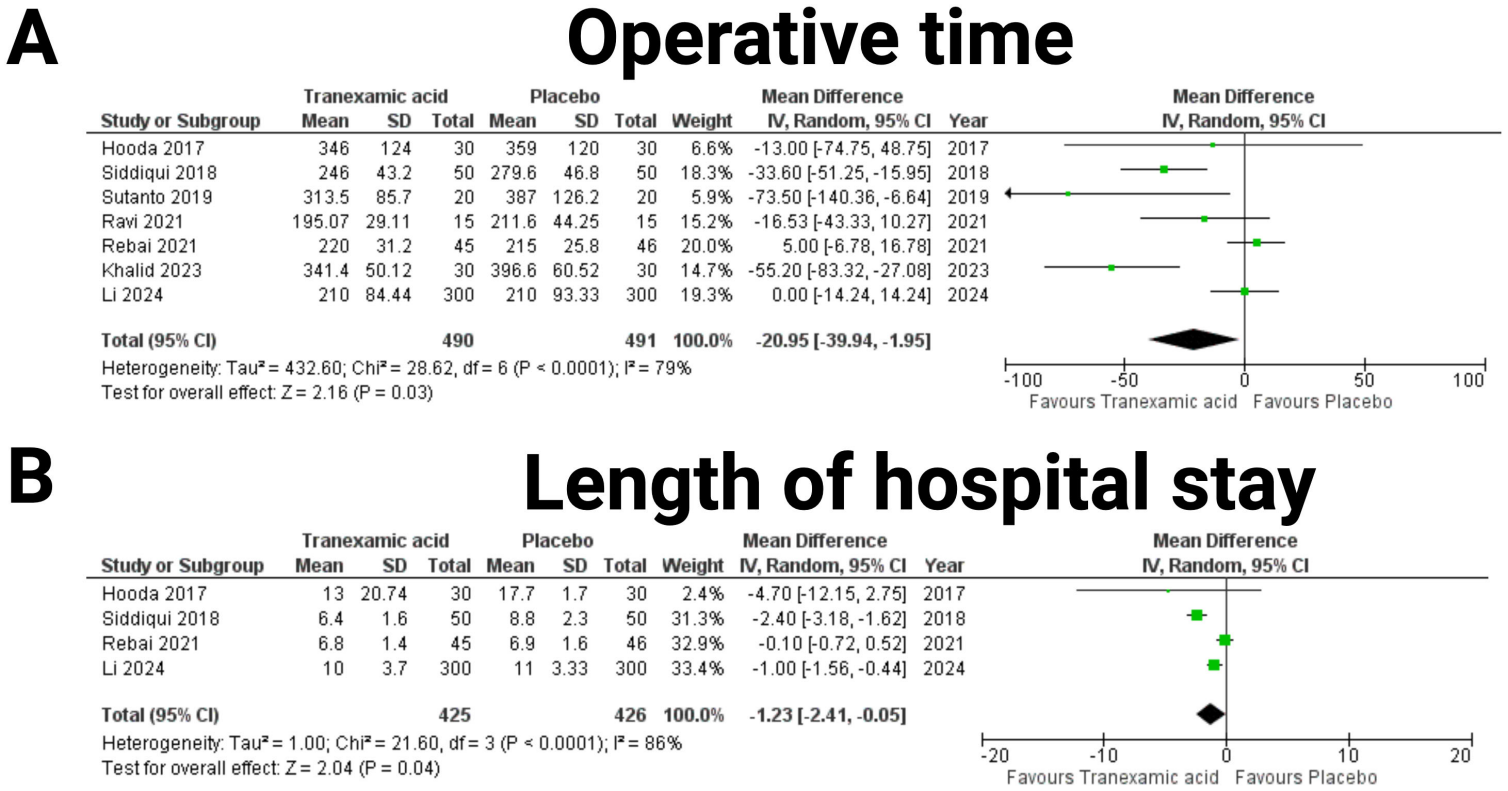
Forest plots illustrating the effect of tranexamic acid versus placebo operative time **(A)** and length of hospital stay **(B)**. Each panel displays mean difference (MD) with 95% confidence intervals (CIs) for individual studies and the combined estimate.

The analysis of operative time included data of 981 patients from seven studies (18–24) (see **Figure 4A**). The combined mean difference was -20.95 minutes (95% CI: -39.94 to -1.95 minutes), indicating a significant reduction in operative time with tranexamic acid compared to placebo (*p* = 0.03). The heterogeneity was moderate to high (I² = 79%, *p* < 0.0001). Furthermore, data of 851 patients from four studies were analyzed regarding length of hospital stay (19, 20, 23, 25) (see **Figure 4B**). The pooled mean difference was -1.23 days (95% CI: -2.41 to -0.05 days), showing a significant reduction in the length of hospital stay for patients treated with tranexamic acid (*p* = 0.04). The heterogeneity was high (I² = 86%, *p* < 0.0001).

### 3.6 Postoperative complications

**Figure 5:**
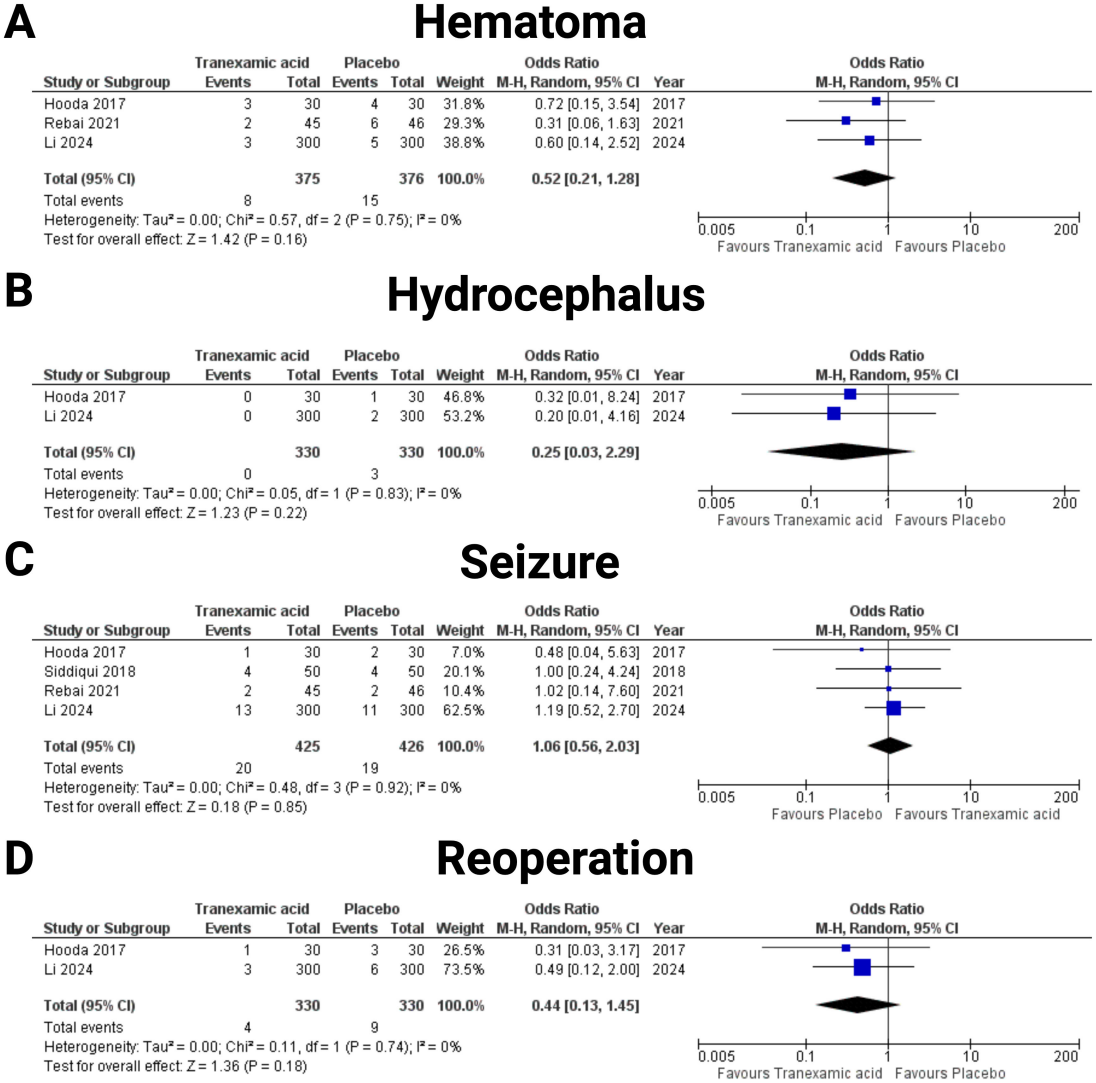
Forest plots illustrating the effect of tranexamic acid versus placebo on various Postoperative complications. Each panel displays the odds ratio (OR) with 95% confidence intervals (CIs) for individual studies and the combined estimate. The following endpoints were investigated: **(A)** Hematoma, **(B)** Hydrocephalus, **(C)** Seizure, and **(D)** Reoperation.

The risk of postoperative hematoma was examined through data from three studies (19, 23, 25). The analysis involved 375 patients in the tranexamic acid group and 376 patients in the placebo group (see **Figure 5A**). The pooled odds ratio was 0.52 (95% CI: 0.21 to 1.28), suggesting a potential reduction in hematoma risk with tranexamic acid treatment, though this finding was not statistically significant (*p* = 0.16). Substantial heterogeneity was not found (I^2^ = 0%, *p* = 0.75). The effect of tranexamic acid on the incidence of hydrocephalus was assessed through two studies (19, 25) (see **Figure 5B**). The analysis included a total of 330 patients in the tranexamic acid group and 330 patients in the placebo group. The pooled analysis yielded an odds ratio (OR) of 0.25 (95% CI: 0.03 to 2.29), suggesting a trend towards a reduction in hydrocephalus among patients treated with tranexamic acid compared to placebo. However, this effect was not statistically significant (*p* = 0.22). The individual study estimates showed considerable variability, with wide confidence intervals crossing the line of no effect, indicating substantial uncertainty in the effect estimate but no statistical heterogeneity was observed (I^2^ = 0%, *p* = 0.22). The incidence of seizures was assessed in four studies (19, 20, 23, 25) (see **Figure 5C**). This analysis included 425 patients in the tranexamic acid group and 426 patients in the placebo group. The meta-analysis resulted in an odds ratio of 1.06 (95% CI: 0.56 to 2.03, I^2^ = 0%, *p* = 0.75), indicating no significant difference in seizure risk between the tranexamic acid and placebo groups (*p* = 0.85). The need for reoperation was evaluated in studies by Hooda et al. (19) and Li et al. (25) (see **Figure 5D**). The total number of patients analyzed included 330 in the tranexamic acid group and 330 in the placebo group. The combined odds ratio was 0.44 (95% CI: 0.13 to 1.45), indicating a non-significant trend towards fewer reoperations in the tranexamic acid group compared to placebo (*p* = 0.18). Significant heterogeneity was not found (I^2^ = 0%, *p* = 0.18). **Figure 5** summarizes the results of postoperative complications. Events of venous thromboembolic complications were only observed in the study by Li et al. (25), which found 26 (26/300, 8.7%) instances in the TXA group, and 22 (22/300, 7.3%), respectively. The other studies by Hooda et al. (19), Ravi et al. (22), Rebai et al. (23), and Siddiqui et al. (20) observed no venous thromboembolic complications in any arm. In the pooled analysis of these five studies with 881 (440 in TXA, 441 in Placebo) patients, TXA was not associated with an increased risk of venous thromboembolic complications in meningioma surgery (OR: 1.18, 95% CI: 0.67- 2.07, I^2^ = 0%, *p* = 0.99).

### 3.7 Physical status at discharge

**Figure 6:**
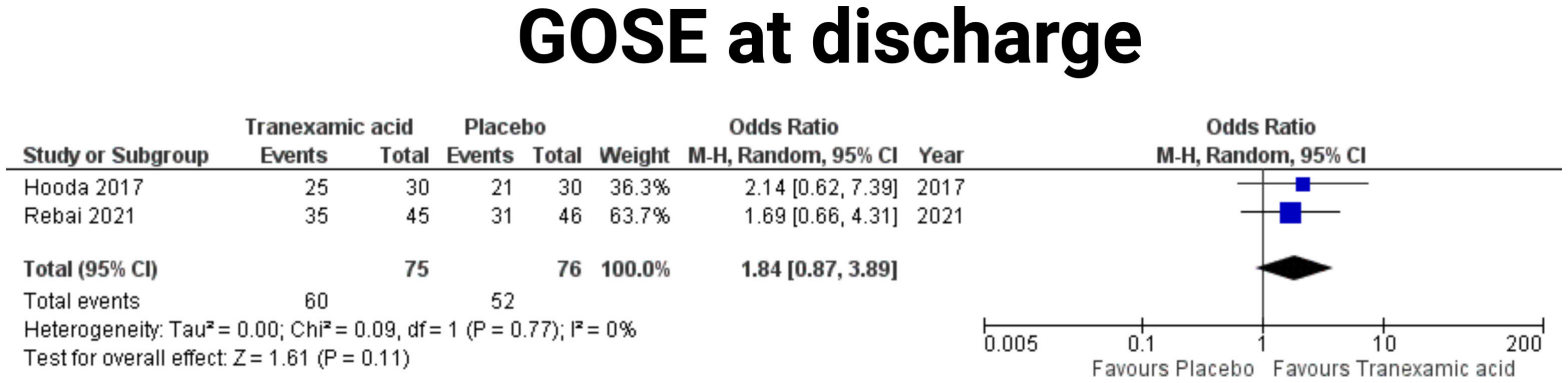
Forest plots illustrating the effect of tranexamic acid versus placebo on GOSE outcome at discharge. Each panel displays the odds ratio (OR) with 95% confidence intervals (CIs) for individual studies and the combined estimate.

A meta-analysis of the effect of TXA on the proportion of patients with good recovery, defined as a Glasgow Outcome Scale Extended (GOSE) score of 7-8 at discharge was performed. Two studies were included in this analysis (19, 23). The pooled analysis included 75 patients in the tranexamic acid group and 76 patients in the placebo group. The combined OR for achieving good recovery with tranexamic acid was 1.84 (95% CI: 0.87-3.89) compared to placebo, indicating a non-significant trend towards improved outcomes with tranexamic acid (*p* = 0.11) (see **Figure 6**). Heterogeneity among the studies was minimal, with an I² of 0%, suggesting consistent findings across the included studies (*p* = 0.77).

### 3.8 Publication bias

**Figure 7:**
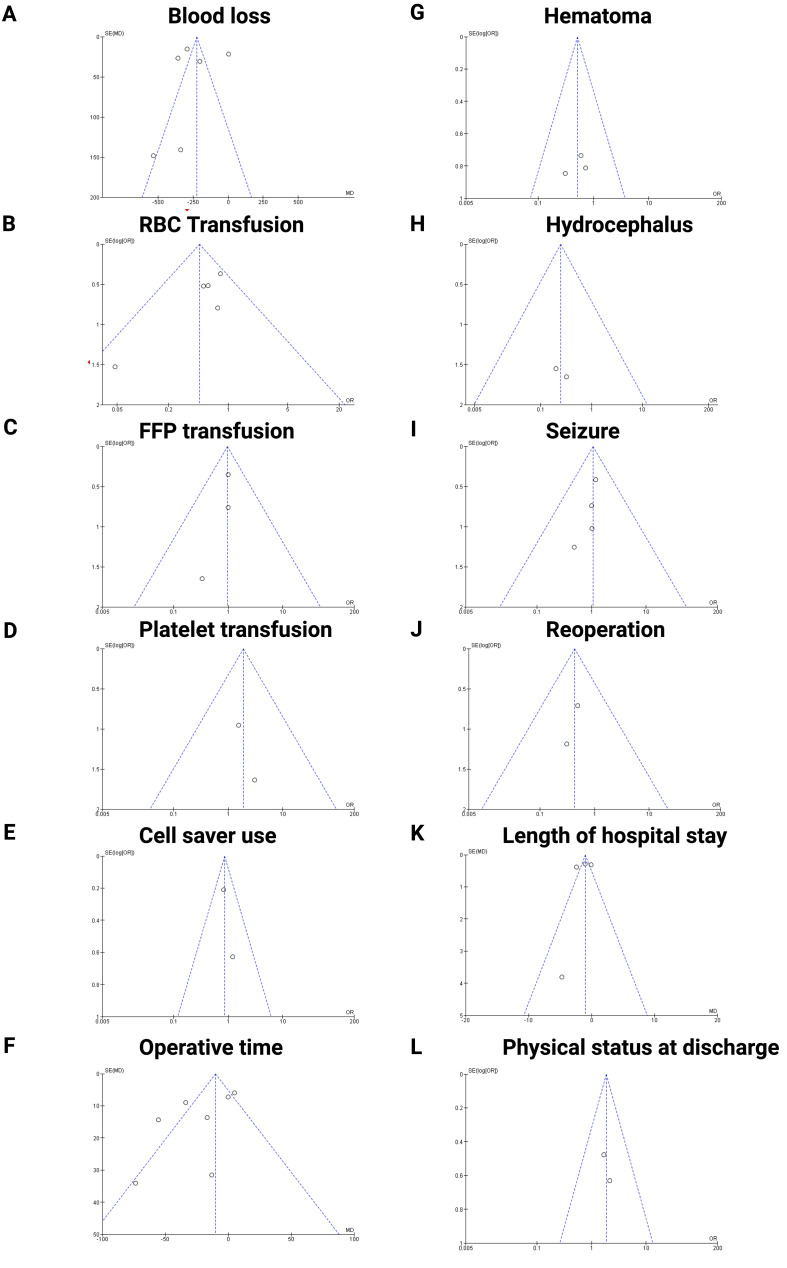
Funnel plots assessing publication bias for the following outcomes: **(A)** Blood loss, **(B)** RBC transfusion, € FFP transfusion, **(D)** Platelet transfusion, **(E)** Cell saver use, **(F)** Operative time, **(G)** Hematoma, **(H)** Hydrocephalus, **(I)** Seizure, **(J)** Reoperation, **(K)** Length of hospital stay, **(L)** Physical status at discharge, respectively. The plots indicate overall symmetry, suggesting minimal publication bias, except for operative time and blood loss which show slight asymmetry.

To ensure acceptable reliability, we implemented three measures to investigate potential publication bias, we employed an extensive literature search strategy. Second, the trials included in this meta-analysis were meticulously selected according to strict inclusion and exclusion criteria. Finally, publication bias was assessed using funnel plots (see **Figure 7**). The majority of the plots exhibited symmetry, with data points scattered evenly around the central line, suggesting minimal publication bias for the majority of outcomes. However, blood loss (**Figure 7A**) and operative time (**Figure 7F**) show slightly asymmetry, suggesting potential publication bias in these outcomes.

## 4 Discussion

We performed a meta-analysis of TXA administration in patients who underwent cranial meningioma surgery. To date, we publish the largest pooled patient collective with 981 patients and 7 eligible prospective randomized and double-blinded controlled trials. The findings of the present meta-analysis can be summarized as follows (see **Supplementary Figure S1** (Graphical abstract)): (1) TXA therapy appears to be associated with reduced blood loss in surgically treated cranial meningiomas; (2) TXA treatment seems to lower the risk of blood transfusions during cranial meningioma surgery; (3) Operative time was significantly enhanced by TXA application; (4) Length of hospital stay was significantly shortened by intraoperative TXA therapy.

### 4.1 Blood loss and transfusion

The present study found a significantly reduced blood loss in cranial meningioma surgery by the use of TXA (MD: -262.7 ml (95% CI: -397.6- -127.8, *p* = 0.0001). Furthermore, TXA use was associated with significantly less blood product transfusions (OR: 0.47; 95% CI: 0.22-0.99; *p* < 0.05). Retrospective cohort of Rajagopalan et al. (5) showed that in the context of elective brain tumor surgery, meningiomas present a significant a risk factor for higher blood loss, which can even overreach the preoperative blood volume. The median of the published blood loss varied between 300 and 1100 ml (26). This very large difference might be due to non-standardized reporting of the underlying pathology, where different types of meningiomas are reported on together without differentiating between WHO grades, meningioma size skull-base vs. non-skull base localization, and vascularization (27, 28). This was also one of the main limitations of the included RCTs, where the subgroup analysis of the meningiomas is not stratified by these parameters regarding blood loss. Despite these limitations, the studies consistently highlight that blood loss can be a significant issue in meningioma surgery and may predict postoperative complications. Although there are inaccuracies in study designs, TXA appears to be a useful tool that can reduce bleeding and shorten operative time when used carefully. The debate around the risks of allogeneic blood transfusions, such as transfusion-related acute lung injury (TRALI), transfusion-associated circulatory overload (TACO), and acute kidney injury, underscores the need to consider patient-specific factors when administering TXA (29). Additionally, the high costs of collecting and administering allogeneic blood products further incentivize the use of effective intraoperative hemostasis strategies.

### 4.2 Complications

While a retrospective study suggested that TXA application increases the risk of venous thromboembolism in surgery for spinal tumors or other high risk patients with active malignancy, this risk profile seems to be not transferrable to TXA application in cranial meningioma surgery (29). The present meta-analysis synthesized 881 individual patient data and found no significant association between TXA and venous thromboembolism in cranial meningioma surgery. This finding might be explained by the mechanism of TXA as a synthetic lysine analog that competitively inhibits lysine-binding sites on fibrin clots to prevent clot lysis, which differs from regular clotting disorders caused by elevated systemic procoagulants relative to anticoagulants, which increases the risk of venous thromboembolism (20).

Irl et al. (30) described the effect of TXA on seizures. In this animal study it has been found that the mechanism of action is inhibition of GABA-receptors in hippocampus, which should lower the epilepsy-threshold and therefore have a strong pro-convulsive effect. The adverse-event database study FAERS also reports on several patients with epileptic symptomatic after TXA administration (31). The present meta-analysis showed that TXA administration is not associated with an increased risk of postoperative seizures (OR: 1.06 (95% CI: 0.56 to 2.03, I^2^ = 0%, *p* = 0.75). However, tumor locations were not stratified in the studies, and it is known that meningiomas at the convexity harbor higher risks of postoperative epilepsy risk compared to skull-base meningiomas (32). Further studies should primarily focus on this anatomical subgroup of meningioma patients.

Other postoperative safety indicators such as hematoma, hydrocephalus, physical status and revision surgery were not significantly influenced by the application of TXA. Nevertheless, it has to be considered that tumor location significantly influences the neurological functioning and postoperative outcome. Meling et al. (33) analyzed 1,148 patients and found that skull base meningiomas (SBMs) are linked to more complex surgeries and a higher risk of postoperative neurological decline, as well as preoperative neurological deficits (RR 1.4; p < 0.0001). Additionally, non-skull base meningiomas (NSBMs) had a higher incidence of seizures (RR 2.2; p < 0.0001) and a greater proportion of WHO grade II and III tumors (10% vs. 4%; p < 0.0001).

### 4.3 Vascularization and hemostasis of meningiomas

Intraoperative blood loss during meningioma surgery varies largely. Against this backdrop, several ongoing research strives to identify predictors of blood loss and management strategies to reduce blood loss. A recent retrospective study of 503 meningioma patients identified tumor area, preoperative albumin concentration, and preoperative platelet count as independent predictors for higher intraoperative blood loss (26). On the other side, other studies also focused on radiological assessment of meningiomas’ vascularity and the meningioma vascularity index based on flow-void volume in T2-weighted sequences has been established to predict blood loss and need for blood transfusion in preoperatively non-embolized meningiomas (34).

Maintaining hemostasis and reducing intraoperative blood loss is crucial for achieving safe maximal resection. Preoperative embolization is another option to reduce blood-loss, which aims to devascularize the lesion. Despite a meta-analysis of matched cohort studies with 434 patients found no significant difference in intraoperative blood loss between those being preoperatively embolized or non-embolized, preoperative embolization resulted in lower odds ratios of major surgically related complications (35). Hence, this procedure might be dedicated for some selective cases and identification of these might be facilitated by the use of arterial spin labeling perfusion MRI (36). TXA as an antifibrinolytic pharmacological means of preventing blood loss demonstrated efficacy in reducing blood loss and blood product usage across various other fields of surgery (37–41). The present meta-analysis investigated blood transfusion requirements in six studies with a total of 841 patients. The studies by Hooda et al. (19), Rebai et al. (23) and Li et al. (25) reported nonsignificant reductions in transfusions, while the studies by Khalid et al. (24), Ravi et al. (22), and Siddiqui et al. (20) showed significant decrease in transfusion requirements among TXA recipients. The reduced blood loss reduces the need for allogeneic blood transfusions, shortens the operative time and length of hospital stay. Hence, TXA administration might also reduce the costs of treatment as suggested in a matched cohort study with patients undergoing primary total hip and knee replacement surgery with or without TXA (42). Further studies will also have to address the economic burden of cranial meningioma surgery with or without TXA application.

### 4.4 Dosing

The current investigation found that TXA use is safe and effective in terms of reducing blood loss, blood transfusions, and facilitating operative time. Despite a high variability with regard to loading doses from 10 to 50 mg/kg, six of the seven included studies used the dose of 20 mg/kg before skin incision (43). This dosage is higher than those reported in orthopedic procedures but within safe limits as established by studies from coronary-artery surgery (44). Against this backdrop, the low complication profile observed in this analysis of 981 patients suggests TXA application as safe and effective.

### 4.5 Limitations & future directions

While the present investigation provides the largest meta-analysis of seven randomized placebo-controlled, and double-blinded trials with 981 patients regarding the use of TXA in cranial meningioma surgery, it is essential to consider some major limitations. The present meta-analysis includes seven studies, which implicates the risk of publication bias. Methods like funnel plot asymmetry might be unreliable for seven studies, making it difficult to draw definitive conclusions (45, 46). All studies in the meta-analysis were conducted outside the US and EU, which may represent a limitation due to geographical concentration. Furthermore, subgroup meta-analysis of outcome parameters by important factors such as age were not possible because the included studies did not stratify into elderly and non-elderly meningioma patients. Range of 95% confidence interval in terms of the analysis of reoperation rates was 1.32 which implicates that the results regarding this endpoint should be interpreted with caution. The indication to perform reoperations in terms of hydrocephalus or postoperative hematoma might also vary between the institutions and influence the outcome analysis. Future research should prioritize refining TXA dosage and administration protocols to enhance efficacy while minimizing risks. Analyzing clinical endpoints in future trials could benefit from focusing on a homogeneous anatomical subgroup of meningiomas, such as non-skull base tumors in order to identify potential functional outcome relevant differences (e.g. GOSE grading, seizure frequency). Additionally, investigating the use of TXA in combination with other hemostatic strategies, like preoperative embolization, could provide valuable insights into optimizing surgical outcomes. By comparing these approaches, researchers can develop more effective treatment protocols that improve patient safety and surgical efficiency in meningioma surgery.

## 5 Conclusion

This meta-analysis demonstrates that intraoperative administration of tranexamic acid (TXA) in cranial meningioma surgery significantly reduces intraoperative blood loss and the need for red blood cell transfusions, thereby shortening operative times and hospital stays. Despite these benefits, TXA does not significantly affect other clinical outcomes such as postoperative seizures, reoperation rates, neurological outcomes, or the incidence of hydrocephalus and hematomas. The safety profile of TXA remains favorable, with no significant increase in the risk of thromboembolic events or other severe complications.

## Data Availability

The original contributions presented in the study are included in the article/**Supplementary Material**. Further inquiries can be directed to the corresponding author.
