# Supplementary figures and images for: Intraoperative tranexamic acid administration in cranial meningioma surgery: a meta-analysis of prospective randomized, double-blinded, and placebo-controlled trials
Source: Front Oncol. 2024 Aug 29;14:1464671. doi: 10.3389/fonc.2024.1464671 (PMC11390351; doi:10.3389/fonc.2024.1464671)

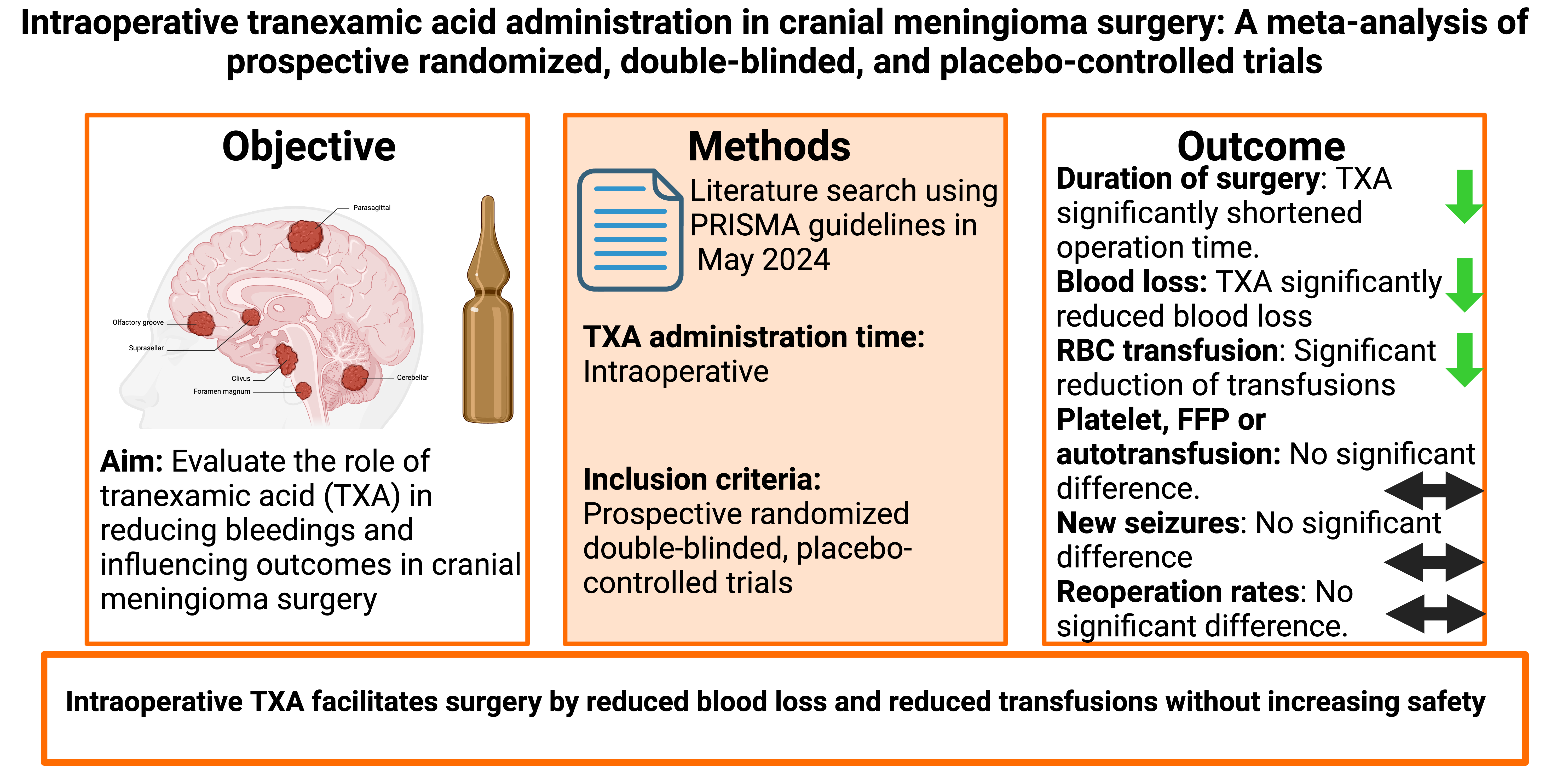

Supplement: Supplementary file 1 [file Image1.tif]
